# Computational Evidence for Bisartan Arginine Blockers as Next-Generation Pan-Antiviral Therapeutics Targeting SARS-CoV-2, Influenza, and Respiratory Syncytial Viruses

**DOI:** 10.3390/v16111776

**Published:** 2024-11-14

**Authors:** Harry Ridgway, Vasso Apostolopoulos, Graham J. Moore, Laura Kate Gadanec, Anthony Zulli, Jordan Swiderski, Sotirios Tsiodras, Konstantinos Kelaidonis, Christos T. Chasapis, John M. Matsoukas

**Affiliations:** 1 Institute for Sustainable Industries and Liveable Cities, Victoria University, Melbourne, VIC 8001, Australia; ridgway@vtc.net; 2 THERAmolecular, LLC, Rodeo, NM 88056, USA; 3 School of Health and Biomedical Sciences, RMIT University, Bundoora, Melbourne, VIC 3083, Australia; vasso.apostolopoulos@rmit.edu.au; 4 Institute for Health and Sport, Immunology and Translational Research Group, Victoria University, Werribee, Melbourne, VIC 3030, Australia; anthony.zulli@vu.edu.au (A.Z.); jordan.swiderski@live.vu.edu.au (J.S.); 5 Pepmetics Inc., 772 Murphy Place, Victoria, BC V6Y 3H4, Canada; mooregj@shaw.ca; 6 Department of Physiology and Pharmacology, Cumming School of Medicine, University of Calgary, Calgary, AB T2N 1N4, Canada; 7 4th Department of Internal Medicine, School of Medicine, National and Kapodistrian University of Athens, 11527 Athens, Greece; sotirios.tsiodras@gmail.com; 8 NewDrug PC, Patras Science Park, 26504 Patras, Greece; k.kelaidonis@gmail.com; 9 Institute of Chemical Biology, National Hellenic Research Foundation, 11635 Athens, Greece; cchasapis@eie.gr; 10 Department of Chemistry, University of Patras, 26504 Patras, Greece

**Keywords:** arginine blockers, bisartans, benzimidazole sartans, coronavirus 2019, influenza, pan-antivirals, respiratory syncytial virus, severe acute respiratory syndrome coronavirus 2

## Abstract

Severe acute respiratory syndrome coronavirus 2 (SARS-CoV-2), influenza, and respiratory syncytial virus (RSV) are significant global health threats. The need for low-cost, easily synthesized oral drugs for rapid deployment during outbreaks is crucial. Broad-spectrum therapeutics, or pan-antivirals, are designed to target multiple viral pathogens simultaneously by focusing on shared molecular features, such as common metal cofactors or conserved residues in viral catalytic domains. This study introduces a new generation of potent sartans, known as bisartans, engineered in our laboratories with negative charges from carboxylate or tetrazolate groups. These anionic tetrazoles interact strongly with cationic arginine residues or metal cations (e.g., Zn^2+^) within viral and host target sites, including the SARS-CoV-2 ACE2 receptor, influenza H1N1 neuraminidases, and the RSV fusion protein. Using virtual ligand docking and molecular dynamics, we investigated how bisartans and their analogs bind to these viral receptors, potentially blocking infection through a pan-antiviral mechanism. Bisartan, ACC519TT, demonstrated stable and high-affinity docking to key catalytic domains of the SARS-CoV-2 NSP3, H1N1 neuraminidase, and RSV fusion protein, outperforming FDA-approved drugs like Paxlovid and oseltamivir. It also showed strong binding to the arginine-rich furin cleavage sites S1/S2 and S2′, suggesting interference with SARS-CoV-2’s spike protein cleavage. The results highlight the potential of tetrazole-based bisartans as promising candidates for developing broad-spectrum antiviral therapies.

## 1. Introduction

The non-structural protein 3 (NSP3) of severe acute respiratory syndrome coronavirus 2 (SARS-CoV-2) contains a crucial enzyme, macrodomain 1 (Mac1), which is common to other viruses (Table 1) and is essential for circumventing host immunity. Recent studies [1] have identified specific compounds that bind firmly to the NSP3 Mac1 of SARS-CoV-2, especially those with anionic characteristics, such as carboxylate or tetrazole groups. This finding raises the possibility that newly developed benzimidazole-based angiotensin receptor blockers (ARBs) and commercially marketed sartans could act as broad-spectrum antiviral medicines against viruses within the *Coronaviridae* family (e.g., SARS-CoV-2, Middle Eastern respiratory syndrome, human coronavirus 229E, and feline coronavirus) [2], in addition to other viruses that contain NSP3, including those within the families of *Hepeviridae* (e.g., hepatitis E virus) and *Togaviridae* (e.g., Chikungunya virus, Semliki forest virus, Sindbis virus, and Venezuelan virus) [3,4,5,6]. In contrast to targeted antiviral therapies, like Paxlovid for SARS-CoV-2, this strategy has the potential to lead to the development of strong binders known as pan-antiviral medicines, such as our newly developed ARB-like bisartans (i.e., benzimidazole *bis*-*N,N′*-biphenyltetrazole (ACC519TT)).

Respiratory syncytial virus (RSV) is an important cause of acute respiratory infection, clinical complications, and death in older adults. After a decade-long quest, the FDA recently approved the world’s first RSV vaccine, Arexvy, which was developed for use in people aged 60 years and older. This vaccine was based on the fusion (F)-protein of RSV, and it has an acceptable safety profile and has been shown to prevent RSV-related acute respiratory infection [7]. However, there is currently no licensed oral antiviral agent against RSV infection. Previous and current research to design and develop oral RSV therapeutics resulted in potential inhibitors of RSV F-protein replication in the preclinical stage (Table 1) [7,8,9,10]. These studies investigated the molecular mechanisms and reported the structures of inhibitors that bound to the RSV F-protein with therapeutic effects. Interestingly, these studies report that the strongest inhibitors were benzimidazole-based compounds exhibiting excellent in vitro potency [9]. Pharmacophoric groups are anionic hydroxylate groups with a nitrile group reminiscent of that of nirmatrelvir, a 3-chymotripsin-like protease (3CLpro) inhibitor, where the nitrile group is the warhead of the antiviral ingredient in Paxlovid [11,12,13,14]. We have created a new class of sartans, referred to as bisartans, composed of a benzimidazole scaffold and bearing two tetrazole/nitriles, which have demonstrated SARS-CoV-2 antiviral abilities in computational, enzyme, and cell culture studies equipotent or greater to those of nirmatrelvir [15,16,17,18,19]. These results also complement clinical studies showing the antiviral abilities of benzimidazole-based ARBs (i.e., candesartan and telmisartan). Thus, we anticipate that our benzimidazole-based sartans, which target both NSP3 (common in influenza and RSV) and the influenza neuraminidase protein (NA), may have broad-spectrum antiviral effects against three viruses: SARS-CoV-2, influenza, and RSV F-protein.

Furin, angiotensin-converting enzyme 2 (ACE2), and 3CLpro are proteases that are involved in spike (S)-protein cleavage, the entry of the S-protein to ACE2, and the replication of the virus [20]. Results from previous studies suggest three possibilities to inhibit coronavirus disease 2019 (COVID-19) (Table 1): (i) the inhibition of S-protein binding to ACE2 (either via the catalytic site or another domain of ACE2) [21]; (ii) the inhibition of viral entry (i.e., furin and transmembrane protease serine 2 (TMPRSS2)) [22]; and (iii) the inhibition of enzymes involved in viral replication or immune escape (i.e., 3CLpro and NSP3) [23]. Furin activates the SARS-CoV-2 S-glycoprotein at characteristic multi-basic recognition sequences, in particular, at the primary subunit (S)1/S2 cleavage site and at the S2′ site, by the trypsin-like protease TMPRSS2 [24,25,26,27,28,29,30]. The cleavage of these viral glycoproteins at the arginine (Arg)-rich multi-basic site subunit (S)1/S2 (680-SPRRARS-686) and multi-basic site S2′ (810-SKPSKRS-816) occurs between the same R-S residues in both sites (685R-686S and 815R-816S, respectively). Thus, Arg blockers (e.g., ARBs and sartans) containing anionic tetrazole and/or carboxyl functional groups and, in particular, bisartans containing two tetrazole moieties with increased acidity represent promising repurposed antiviral drugs [31].

Furin provides a therapeutic target for viral pathogens, including SARS-CoV-2 and bacterial infections [24,32,33]. Crystallography and computational studies have shed light on the role of tryptophan (Trp)254 during furin activity as a molecular gate promoting antiviral drug binding [33,34]. Tested compounds containing tetrazole or carboxyl, such as ACC519TT, exhibit antiviral activity [34]. The arginine (Arg)-rich sequence 681–684 (PRRA) at the S-protein S1/S2 site enables cleavage by furin but not in SARS-CoV, which lacks this sequence [25]. Sartans, as anionic compounds, can interact with positive Args of the S1/S2 and S2′ cleavage sites and prevent cleavage, which is favored by the presence of Arg residues that catalyze a scissoring reaction. As previously mentioned, our novel class of benzimidazole sartans, which bear anionic hydroxylates, tetrazoles, or carboxylic groups, demonstrate dual antihypertensive and antiviral actions against COVID-19 [15,35]. This class is structurally related to other commercially available ARBs, such as telmisartan and candesartan, which also contain a benzimidazole scaffold, have been found to show beneficial effects in SARS-CoV-2 patients [31,36,37], and are comparable to the main antiviral drug nirmatrelvir, where nitrile is the warhead pharmacophore group [15,18]. In another approach, a novel class of furin inhibitors containing a 3,5 dichloromethyl pyridine moiety were recently reported [33]. Intriguingly, these molecules disturb the catalytic charge relay system triad (i.e., aspartic acid (Asp)153, histidine (His)194, and serine (Ser)368) of furin proven to be highly potent at nM concentrations and highly effective in vivo [34]. Results based on crystallography revealed that furin’s catalytic sites tryptophan (Trp)254 and His194 undergo major dihedral shifts upon ligand binding [34]. In this model, the Asp153 residue of the triad interacts with piperazine of the inhibitor and Trp254 interacts with the dichlorophenyl moiety. Thus, drugs in the sartan family, including ACC519TT [34], can block furin activity (and subsequent infection) by obstructing the basic amino acids of cleavage sites [15,16,17].

The discovery of ACE2 almost two decades ago as the principal component of the non-classical arm of the renin–angiotensin system (RAS) has opened new avenues for effective therapies in cardiovascular diseases (CVDs) and COVID-19 [38,39,40,41,42]. Clinical studies have shown the beneficial effect of ARBs, telmisartan, candesartan, and losartan in hypertensive patients infected with SARS-CoV-2 compared to patients not taking ARBs [31,36,37]. The interaction of sartans with the ACE2 receptor-binding domain (RBD) complex by computational and enzyme studies and the role of warhead tetrazole has been recently reported [18]. In these studies, the interactions of tetrazole-containing sartans with ACE2 were experimentally validated by surface plasmon resonance (SPR) analyses with recombinant human ACE2 protein. Bisartans with two tetrazoles were found to be the best binders with ACE2 compared to sartans with one tetrazole [18].

Influenza viruses (A, B, and C) contain the virion surface glycoprotein NA, which is the main target for neutralizing antibodies and for the design of antiviral inhibitors (Table 1). The enzymatic site of NA contains a cluster of Args (i.e., Arg118, Arg152, Arg224, Arg292, and Arg371) bound to sialic acid. Inhibition can occur by preventing salt-bridge formation between the carboxylate of sialic acid and the Args. Other targets are tyrosine (Tyr)406, which functions as a catalytic nucleophile of NA and, similarly, as a charge relay system nucleophile in the Ser and cysteine proteases, and calcium, which stabilizes the crustal active structure of NA bound to the sialic acid through Args [43]. FDA-approved influenza drugs, such as zanamivir, oseltamivir, and peramivir, were compared by crystallographic docking with our benzimidazole-based bisartans. Thus, determining if our bisartans can target NA will shed light on their pan-antiviral abilities.

Bisartans have the potential to be effective against respiratory infections in two ways: (i) by reducing the negative effects of pathogenic RAS components (e.g., angiotensin II (AngII)), such as pulmonary edema, inflammation, and cytokine storm, and (ii) by blocking interactions between the ACE2 receptor and the S-protein of the virus. This interference ultimately prevents the virion from entering the host cells and is particularly important for the receptor and zinc active sites. ARBs, bisartans, and other Arg blockers with anionic tetrazoles and carboxylates show promise as prospective antiviral candidate medicines, according to in silico studies, by reducing the affinity of the S-protein’s receptor-binding domain for ACE2 and inhibiting the hydrolysis of cleavage sites [15,44,45,46]. In the current study, we evaluated bisartans and benzimidazole-based sartans as antivirals by investigating their interactions with components of SARS-CoV-2 (e.g., furin, NSP3, Mac-1, and Papain-like protease (PLpro)), RSV (e.g., F-protein), and influenza viruses (e.g., NA) using ligand–receptor docking studies and molecular dynamics (MD) simulations.

## 2. Materials and Methods

Protein structures were extracted from the PDB files and prepared for docking by removing co-crystal ligands using PyMOL (Verison 2.6.0). Any bound water molecules or heteroatoms were also eliminated. The cleaned protein structures were then saved in a suitable format for docking simulations. Ligand–receptor docking was performed using AutoDock VINA (v1.2.5) with default parameters as implemented in the YASARA Dynamics suite (Yet Another Artificial Scientific Reality Application: http://www.yasara.org/) (accessed in 1 May 2024) [47]. Point charges and dihedral barriers were assigned based on the AMBER14 force field [25,48]. Partial atomic charges were damped to mimic less polar Gasteiger charges for optimal AutoDock scoring function [49]. The best hits and ligand conformational poses, represented as kcal/mol free energy of binding, were reported based on a minimum of 100 but typically 900 runs per ligand.

MD simulations were conducted using the YASARA Dynamics software (version 24.4.10) [47,48]. The simulation setup involved optimizing the hydrogen bonding network to enhance solute stability and predicting the pKa values to determine the protonation states of protein residues at pH 7.4. NaCl ions were added at a physiological concentration of 0.9%, with an excess of either Na or Cl ions to neutralize the system. After steepest descent and simulated annealing minimizations to eliminate clashes, the MD simulation was run for a maximum of 120 ns. The AMBER14 force field [26] was employed for the solute, while General AMBER force field 2 and semi-empirical with bond charge correction were used for ligands and “transferable intermolecular potential with 3-points” for water molecules. The van der Waals forces were calculated with a cutoff of 8 angstrom (Å), following the default settings of AMBER14. Electrostatic forces were computed using the mesh Ewald algorithm, and no cutoff was applied. The equations of motion were integrated with multiple timesteps of 2.5 fs for bonded intermolecular interactions and 5.0 fs for non-bonded interactions. The simulations were performed at a temperature of 311 °K and a pressure of 1 atm in the NPT ensemble, utilizing algorithms previously described in detail [50]. Ligand binding energy was calculated using two methods. First, the ∆G of ligand–protein binding was estimated with AutoDock VINA, incorporating entropic and desolvation effects. Second, in MD simulations, binding free energy (∆ Gbinding) was calculated by removing water for each frame analysis and applying an implicit solvation model accounting for solvation energy and the entropic costs of solvent exposure. RMSD values were calculated using the “YASARA Structure” software (version 24.4.10) (Yasara.org) (accessed 1 May 2024), with measurements taken for all heavy atoms in the protein and ligands.

## 3. Results

### 3.1. Docking of ACC519TT and Other Ligands to SARS-CoV-2 NSP3 Mac1

The COVID-19 pandemic, though reduced in severity, remains a global health issue, with over 750 million infections and nearly 7 million deaths by early 2023 [51]. Current antivirals are limited, underscoring the need for new treatments [52]. SARS-CoV-2, a positive-stranded RNA virus, encodes NSP3, which includes the Mac1 domain with ADPR-ribosylhydrolase activity, affecting viral replication and immune response [53,54,55,56,57,58,59,60,61,62,63,64]. Recent findings show that mutations in Mac1 can drastically reduce its activity and viral replication [65,66].

**Figure 1:**
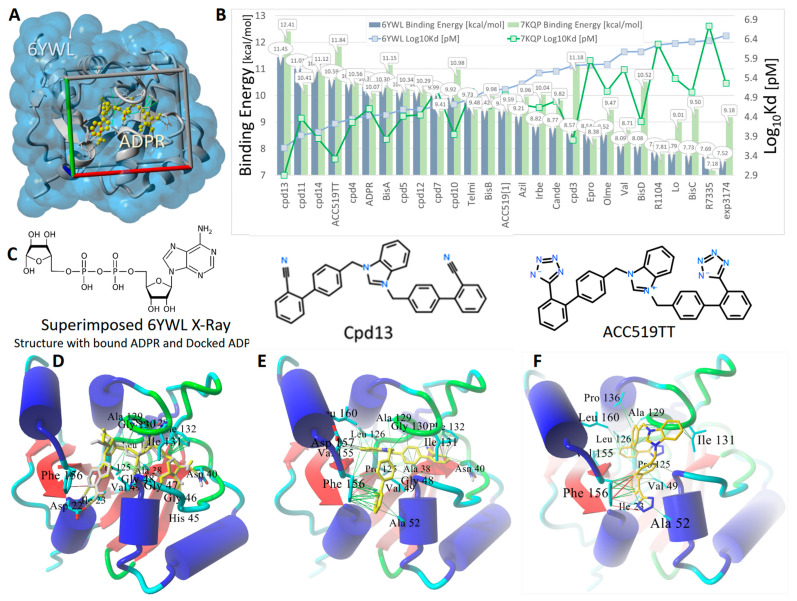
Docking of experimental sartans (e.g., ACC519TT, Cpd13, BisA, etc.), FDA-approved sartans (e.g., candesartan, olmesartan, losartan, etc.), and known inhibitors (e.g., R1104 and R7335) of the SARS-CoV-2 NSP3 Mac1 domain. (**A**) Overview of the docking setup showing the X-ray crystallographic structure for the SARS-CoV-2 Mac1 domain, PDB 6YWL, rendered as gray ribbons, and the water-accessible surface (blue shading) with its bound native ligand, ADPR (yellow atoms as spheres). The docking region of interest in which energy grids were constructed is indicated by the walled “periodic” box with colored lines of dimensions (x/red = 26 Å, y/green = 22 Å, and z/blue = 20 Å). (**B**) Docking results for 27 selected ligands targeting the NSP3 Mac1 domain of SARS-CoV-2. Docking was carried out against two PDB crystallographic structures: 6YWL (blue bars) and 7KQP (light green bars). Ligand docking was performed using AutoDock VINA with AMBER14 force field point charges and dihedral barriers (900 runs per ligand). Docking results are expressed as ligand binding energies (kcal/mol) and calculated dissociation constants (Log10Kd in pM units). Of the 27 docked ligands, Cpd13, a di-phenylcyano-derivative of the anionic bisartan ACC519TT, exhibited the strongest Mac1 binding at 11.45 and 12.41 kcal/mol for the 6YWL and 7KQP Mac1 receptors, respectively. Compared to ACC519TT binding (10.59 and 11.84 kcal/mol, respectively, for binding to the 6YWL and 7KQP receptors), ADPR, which is the native ligand for the Mac1 domain, exhibited somewhat weaker binding (10.32 and 10.07 kcal/mol, respectively, for 6YWL and 7KQP). Surprisingly, compounds R1104 and R7335, which are experimentally proven inhibitors of the NSP3 Mac1 domain [52], exhibited poor binding energies compared to nearly all the FDA-approved and experimental sartans. (**C**) Structures of ADPR, the di-phenylcyano-(bisartan)-derivative Cpd13, and bisartan ACC519TT. Chemical key: H, hydrogen; N, nitrogen; O, oxygen; P, phosphorus. (**D**) Docking validation for ADPR: Docked ADPR pose (green C atoms as spheres) in the Mac1 receptor superimposed onto the 6YWL X-ray structure with bound ADPR (cyan C atoms as spheres). RMSD for the superimposed protein-ligand complexes was ≤ 0.0001 Å. These data indicate that AutoDock VINA was able to accurately calculate the correct X-ray pose for this complex ligand. ADPR was stabilized in the Mac1 domain by approximately six hydrogen bonds (thick yellow dashed lines), as well as pi–pi (red lines) and hydrophobic interactions (green lines). (**E**) Binding mechanism of Cpd13 (di-phenylcyano-derivative of ACC519TT) in the NSP3 Mac1 domain. The docked ligand was stabilized mainly by ionic pi–cation interactions (thin red lines) between one of the terminal phenylcyano groups and Mac1 residue Phe132. The other phenylcyano group entered hydrophobic interactions (thin green lines) with Phe156 and Ala52. Phe156 also was bonded to the phenyl group adjacent to the benzimidazole group of Cpd13 by pi–cation interactions (thin red or magenta lines). (**F**) Binding of ACC519TT (yellow C atoms rendered as tubes) in the Mac1 pocket involved numerous hydrophobic interactions (green lines) between the phenyl groups of ACC519TT and residues Ala52, Ile131, Ala129, Pro136, Leu160, Leu126, Val155, Val49, Ile23, and Phe156. Additional pi–pi interactions (red line) were observed between Phe156 and one of the phenyl groups of ACC519TT. Abbreviations: ACC519TT, benzimidazole bis-N,N’-biphenyltetrazole; ACC519T[1], benzimidazole-N-biphenyltetrazole; ADPR, adenosine 5′-diphosphoribose; Ala, alanine; AMBER, Another Model Building Energy Refinement; Asn, asparagine; Asp, aspartic acid; Azil, azilsartan; Bis, bisartan; Cande, candesartan; Epro, eprosartan; EXP3174, Gly, glycine; Irbe, irbesartan; Ile, isoleucine; Leu, leucine; Lo, losartan; Mac1- macrodomain-1; NSP3, non-structural protein 3; Olme, olmesartan; PDB, Protein Data Bank; Phe, phenylalanine; Pro, proline; SARS-CoV-2, severe acute respiratory syndrome coronavirus 2; Ser, serine; Telm, telmisartan; Val, valine; Å, angstrom.

The above observations illustrate the critical role of Mac1 in promoting viral replication and pathogenesis by down-regulating host innate immunity and strongly suggest Mac1 as a viable therapeutic target. Here, we describe preliminary molecular docking results comparing the binding energies and calculated dissociation constants for a group of FDA-approved antihypertensive sartans, experimental bisartans, and several known Mac1 inhibitors targeting the NSP3 Mac. Docking was performed against two Mac1-domain crystallographic models, Protein Data Bank (PDB) 6YWL and 7KQP, which shared 100% sequence identity but differed in their side chain rotomer distributions (RMSD = 0.304 Å over 163 residues following alignment using Multiple Structural Alignment Algorithm [51]) (Figure 1). Results from these docking studies indicated that of the 27 docked ligands, Cpd13, a di-phenylcyano-derivative of the anionic bisartan ACC519TT, exhibited the strongest Mac1 binding energy at 11.45 and 12.41 kcal/mol for the 6YWL and 7KQP Mac1 receptors, respectively. The slight difference in ligand binding between 7KQP and 6YWL likely results from their variations in crystallization conditions and distinct crystal forms, which can affect protein conformation and binding site accessibility. Compared to ACC519TT binding (10.59 and 11.84 kcal/mol, respectively, for the 6YWL and 7KQP receptors), ADP, which is a native substrate for the Mac1 domain, exhibited somewhat weaker binding (10.32 and 10.07 kcal/mol, respectively, for 6YWL and 7KQP). The superimposition of the 6YWL X-ray crystallographic structure with bound ADPR onto the receptor with docked ADPR yielded an RMSD value of <0.0001 Å, indicating that the Autodock VINA algorithms were able to accurately predict a correct conformational pose for this ligand.

Surprisingly, compounds R1104 and R7335, which are experimentally proven inhibitors of the SARS-CoV-2 NSP3 Mac1 domain [52], exhibited substantially weaker binding energies (approximately 7.5 kcal/mol) compared to nearly all FDA-approved and experimental sartans, with the possible exceptions of BisC and BisD. In several instances, ligand binding was significantly stronger for the 7KQP Mac1 domain compared to that for 6YWL; however, the reason for this discrepancy was not investigated as part of this study.

### 3.2. Docking to the NSP3 PLpro Domain of SARS-CoV-2

The SARS-CoV-2 polyproteins pp1a and pp1ab are cleaved into 16 NSPs by the viral PLpro and the 3CLpro. The latter is also referred to as the main protease (Mpro). PLpro cleaves NSP1, NSP2, and NSP3 at its LXGG recognition sites [53], whereas Mpro cleaves the remaining downstream NSPs (NSP4-16) [53,54]. Because they are involved in the earliest stages of viral infection and replication, Mpro and PLpro have been the subjects of intensive research into antiviral therapeutics [53,54,55].

**Figure 2:**
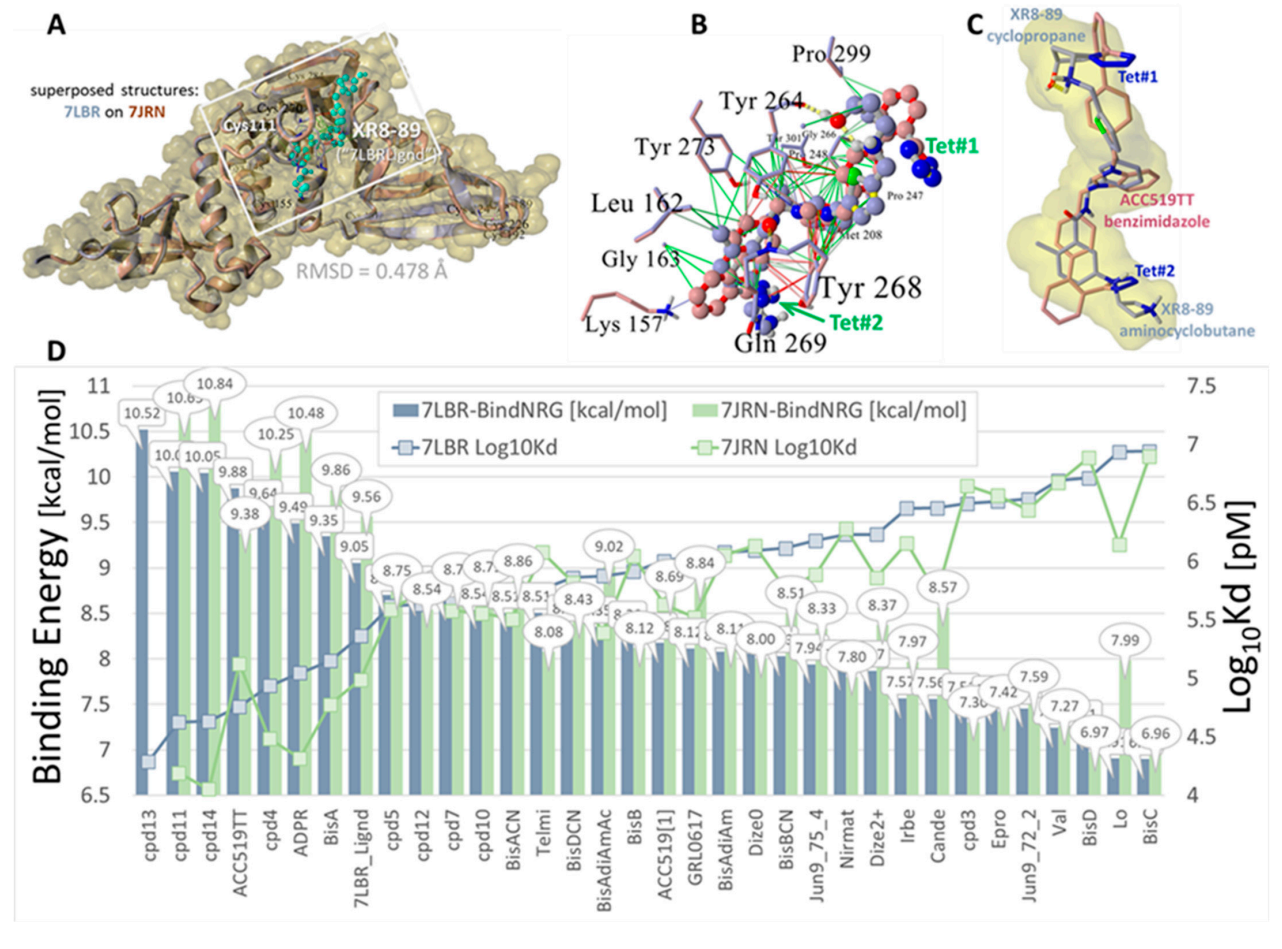
Docking of selected ligands to the SARS-CoV-2 NSP3 PLpro (PDB: 7LBR and 7JRN). Experimentally proven drugs investigated include PLpro inhibitors (i.e., XR8-89 [56]; GRL0617, Jun9-72-2, and Jun9-74-4 [14]). **Upper panel**, (**A**): The 7LBR PLpro domain X-ray crystallographic structure (blue ribbons) superimposed onto PLpro 7JRN (maroon ribbons). Overall RMSD for aligned structures = 0.478 Å. Approximate boundaries of the docking region, which contained the catalytic Cys11 residue, are indicated by the gray rectangle. The 2-phenylthiophene-based inhibitor “7LBRLignd” (XR8-89) [56] bound in the “BL2” groove proximal to the catalytic site is also indicated (cyan atoms). The 7LBR structure has been rendered as the Van der Waals surface (yellow shading). **Upper panel**, (**B**): Docked bisartan ACC519TT (dusty blue carbon atoms) superimposed on the docked PLpro inhibitor XR8-89 (maroon carbon atoms) for the 7LBR receptor. ACC519TT adopted a conformation along the BL2 groove that was similar to XR8-89 (molecule pair RMSD = 9.49 Å), with both ligands sharing a number of close contacts with 7LB6 residues, including Leu162, Tyr273, Tyr264, Pro299, Tyr268, and Gln269. Non-bond drug–receptor interactions included hydrophobic (green lines), pi–pi resonance (red lines), cation–pi (blue to light-blue lines), and hydrogen bonds (dashed yellow lines). Locations of the dual anionic tetrazole groups are labeled in blue as Tet#1 and Tet#2. **Upper panel**, (**C**): The bisartan tetrazole functionalities appeared bioisosteric with the terminal aminocyclobutane and cyclopentane groups of XR8-89. A similar relationship was observed for the central benzimidazole group of ACC519TT that overlapped the central benzene ring of XR8-89. **Lower panel**, (**D**): Docking results expressed as ligand binding energies in kcal/mol. Log_10_Kd values in pM units were computed from the binding energies [32]: color key: blue bars = docking to the PLpro domain of PDB 7LBR and green bars = docking to the PLpro domain of PDB 7JRN. Abbreviations: ACC519TT, benzimidazole *bis*-*N,N′*-biphenyltetrazole; ACC519T[1], benzimidazole-*N*-biphenyltetrazole; Azil, azilsartan; Bis, bisartan; Cande, candesartan; cpd, compound; DIZE, diminazene aceturate; Epro, eprosartan; Gln, glutamine; Gly, glycine; Irbe, irbesartan; Leu, leucine; Lo, losartan; Mac1, macrodomain-1; Met, methionine; Nirmat, nirmatrelvir; NSP3, non-structural protein 3; Olme, olmesartan; PLpro, papain-like protease; Pro, proline; RMSD, root-mean-standard deviation; RSV, respiratory syncytial virus; SARS-CoV-2, severe acute respiratory syndrome coronavirus 2; Tyr, tyrosine; XR8-89, 7:BR-Ligand; Å, Angstrom.

Here we compare the PLpro docking profiles of several recently experimentally proven PLpro inhibitors (e.g., XR8-89 [56]) to those of antihypertensive bisartans, sartans, and related compounds (Figure 2). For comparative purposes, docking was carried out targeting two PLpro X-ray crystallographic structures, including 7LBR and 7JRN, which shared 100% sequence identity but differed by slight variations in their sidechain rotomer distributions (overall RMSD = 0.478 Å) [56]. As shown in Figure 2, ligand docking results were similar for both receptors, consistent with minor variations in their sidechain conformers. Note that the “7LBRLigand” (alias XR8-89) is a 2-phenylthiophene-based non-covalent PLpro inhibitor designed specifically for the 7LBR target [56], and it was only docked against the 7LBR receptor in our study. Not surprisingly, this ligand exhibited slightly stronger binding to 7LBR compared to the bisphenyltetrazole ACC519TT, and both ligands exhibited superior binding scores compared to the positive control (inhibitor) GRL0617 [56].

### 3.3. Docking to the RSV F-Protein

**Figure 3:**
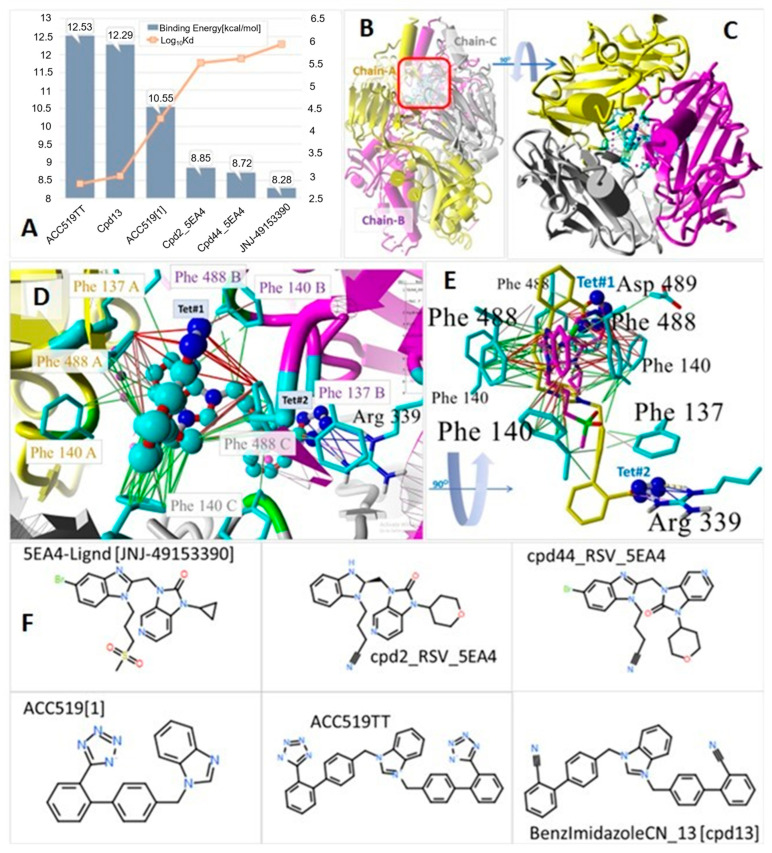
(**A**) Docking results of selected ligands targeting the RSV F-postfusion (i.e., drug-induced) protein that mediates virus entry into host cells. In its native (host-free) state, the homotrimeric F-protein exists in a metastable “prefusigenic or prefusion” conformation and must undergo a structural rearrangement that facilitates membrane fusion [61]. Ligands were docked into the three-fold symmetric domain (3FSD) of the virion F-protein (PDB 5EA4) located in the upper (surface) central cavity where the three chains intersect, denoted in the side-view projection by the red square in (**B**). Induced-fit binding of the potent RSV F-protein inhibitor JNJ-49153390 within the 3FSD interlocks two of the protomers in the pocket, effectively stabilizing the prefusion conformation and preventing host cell fusion and infection. Docking results indicated the bisartan ACC519TT bound significantly more strongly (12.53 kcal/mol) into the 3FSD pocket compared to all other drugs tested. The binding energy of JNJ-49153390 (8.28 kcal/mol), as well as those of two other structurally similar experimentally proven F-protein inhibitors (i.e., cpd2-5EA4 and cpd44-5EA4) were substantially lower. (**C**) Structure of 5EA4 with docked ACC519TT (in the drug-bound postfusogenic conformation) showing the three color-coded protomers (Key: yellow = Chain-A; magenta = Chain-B; gray = Chain-C) in the down-axis view rotated 90° from the side view in B. (**D**) Magnified down-axis view from C showing binding mechanism of ACC519TT involving a putative tethering of all three protomers by interactions with symmetrically arranged 5EA4 residues Phe488 and Phe140 (in each chain). Unlike the binding of JNJ-49153390, ACC519TT binding also involved strong electrostatic (salt bridge/pi–cation) interactions (blue lines) of the tetrazole#2 (Tet#2) functional group with a deeply buried Arg339 residue in Chain-B. The tetrazole#1 (Tet#1) group of ACC519TT was effectively coordinated by two of the symmetrically arranged phenylalanine residues (Phe488-A and Phe488-C) through pi–pi resonance bonding (red lines). This type of dual protomer binding by ACC519TT was similar to that reported by Battles and coworkers [61] regarding JNJ-49153390. Finally, additional hydrophobic interactions (green lines) between Phe140-C and one of the phenyl groups proximal to Tet#1 and adjacent to the central benzimidazole moiety also contributed to ACC519TT stability in the 3FSD pocket. (**E**) Side-view image rotated 90° from D showing docked ACC519TT (yellow C atoms) superimposed onto the X-ray crystallographic pose of the F-protein antagonist JNJ-41953390 (magenta C atoms). This view illustrates more clearly the interaction of Tet#2 with the buried Arg339 residue of Chain-B through ionic (blue lines) and hydrogen bonding (thick dashed yellow line). (**F**) Chemical structures of the six ligands evaluated. Chemical key: O. oxygen; S, sulfur; Br, bromine; N, nitrogen. Abbreviations: ACC519TT, benzimidazole *bis*-*N,N′*-biphenyltetrazole; ACC519T[1], benzimidazole-*N*-biphenyltetrazole; Asp, aspartic acid; Arg, arginine; cpd, compound; F-protein, fusion protein; PDB, Protein Data Bank; Phe, phenylalanine; RSV, respiratory syncytial virus; S, sulfur; Tet, tetrazole; JNJ49153390, 5EA4-Ligand; 3FSD, 3-fold-symmetric domain.

RSV is a ubiquitous paramyxovirus that is a leading cause of acute lower respiratory infections in children (<5 years) and the elderly [57,58]. Despite much research to date on RSV, there remains a pressing need for cost-effective interventions [8]. RSV possesses two major glycoproteins on its surface: the attachment glycoprotein (G) and the fusion (F) glycoprotein (see Figure 3B). These proteins control the initial attachment and infection phases of the virion [59]. The G protein is not absolutely required for infection since deletion mutants only result in attenuation [60]. It is the F-protein that facilitates the virion membrane to fuse with a host cell membrane. Prior to cell fusion, the RSV F-protein exists in a metastable “prefusional or prefusigenic” three-fold symmetric conformation that must undergo incompletely understood structural rearrangements needed to invoke cell fusion. However, by blocking such rearrangements with drugs, it is possible to impede or completely prevent viral infection. For example, Battles and coworkers [61] reported the use of several experimental and therapeutically active drugs, including a butylsulfonyl-derived drug, JNJ-49153390, which stably binds to the three-fold symmetric surface domain (3FSD) of the F-protein [61]. The drug’s mechanism of action involves a localized induced-fit rearrangement of the three intersecting F-protein protomers in which two of the protomers become irreversibly tethered via pi–pi interactions by the bound drug, thereby precluding the transition of the F-protein from the prefusigenic conformation.

Here, we describe the results of docking studies in which the binding energies of three sartan drugs (including the antiviral/antihypertensive bisartan ACC519TT) are compared to those of three drugs specifically designed to target the RSV F-protein, including inhibitor JNJ-49153390 [61]. The docking results, which are summarized in Figure 3, indicate that bisartan ACC519TT exhibited the strongest binding in the surface 3FSD F-protein pocket. The binding motif of ACC519TT was similar to that of JNJ-49153390, since both entered strong pi–pi and electrostatic interactions that tied the F-protein protomers together. However, due to its longer length (compared to JNJ-49153390) and additional torsional flexibility, the former (bisartan) was also able to undergo a strong pi–cation interaction with a deeply buried cationic Arg339 residue in Chain-B (see Figure 3 for more details). Based on these observations, it is reasonably speculated that ACC519TT may outperform some of the existing experimental RSV designer drugs that have been shown to have therapeutic benefits in animal studies [62].

### 3.4. Docking of Bisartan ACC519TT with S1/S2 and S2′ SARS-CoV-2 FCSs

A common motif for viral entry into host cells and subsequent replication involves the fusion of the viral membrane envelope with the host cytoplasmic membrane. Membrane fusion is mediated by thermodynamically favorable conformational rearrangements of viral entry protein(s) from an energetically stable pre-infective status to a metastable configuration predisposed to conversion to a lower-energy state enabling membrane fusion. In SARS-CoV-2 and related coronaviruses, the spike glycoprotein transition is catalyzed following ACE2 engagement by sequential proteolytic cleavage steps involving the multi-basic S1/S2 consensus sequence site (Arg-Arg-Ala-Arg) and a second S2′ site in the S2 subunit [40]. Cleavage at S1/S2 is a prerequisite for proteolysis at S2′, and both actions are essential to initiate membrane fusion. Cleavage at these sites is mediated by furin-like proteases, including host TMPRSS2, localized on the cell surface and/or cathepsin-L confined to endolysosomes. The cleavage of the S2′ site exposes the fusion peptide (FP), and the dissociation of S1 from S2 triggers a cascade of rapid structural changes in the S2 subunit, especially in heptad repeat 1, forcing the FP into the target plasma membrane, thus initiating fusion. If TMPRSS2 expression is insufficient or the virus/ACE2 complex fails to encounter TMPRSS2, ACE2-bound virus may be internalized via clathrin-mediated endocytosis [63,64], forming an endolysosome where S2′ is cleaved by non-specific host cathepsin-L. Regardless of the mode of viral entry into host cells, the involvement of the S1/S2 and S2′ sites early in the infection process makes them promising targets for the development and optimization of small-molecule antiviral drugs.

**Figure 4:**
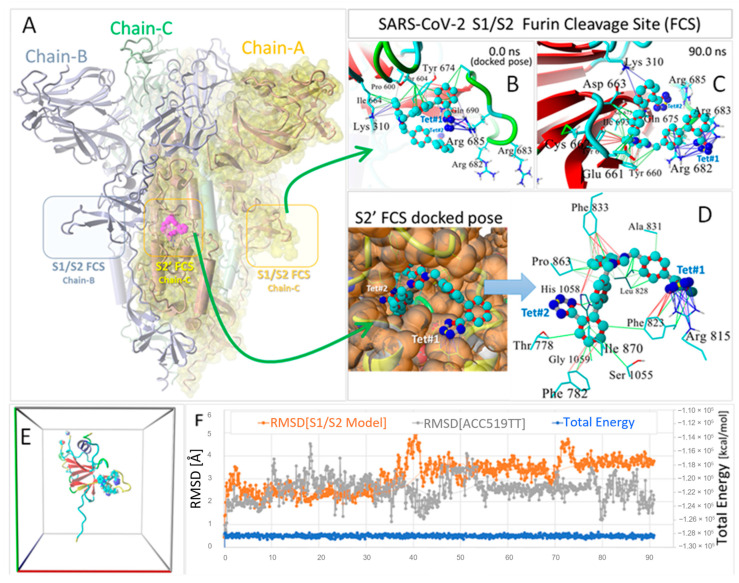
Binding of bisartan ACC519TT to the furin cleavage sites of the SARS-CoV-2 spike (S) protein. (**A**) Full-sequence homology model (Swiss Model 05) of the SARS-CoV-2 spike protein showing locations of the S’ and S1/S2 FCSs. The three homotrimeric chains are color coded: Chain-A = yellow with Van der Waals surface (yellow shading); Chain-B = green; Chain-C = blue. The model is rotated so that the S2′ FCS of Chain-A with docked bisartan ACC519TT (cyan carbon atoms) is shown located in the center of the model. (**B**) Docked pose of bisartan ACC519TT in the S1/S2 spike FCS consensus loop region showing the interaction of tetrazole#1 (Tet#1) with Arg685. (**C**) ACC519TT conformation following 90 ns of an NPT MD simulation at 311 °K, 0.9%wt/vol saline with periodic boundaries (see Methods) of an isolated model “fragment” of the S1/S2 FCS binding domain depicted in (**E**). Water and NaCl ions have been hidden for clarity. The four terminal fragment residues of the FCS model in (**E**) were capped and frozen during the MD simulation. Analysis of the MD trajectory indicated the total system energy was essentially equilibrated throughout the simulation (blue line in (**F**)). Despite the significant thermal motion of the FCS model (RMSD ranged from about 1 to 4 Å), the bisartan remained stably bound for the 90 ns duration of the MD simulation (drug RMSD ranged from about 1 to 5 Å). The comparison of the initial docked drug pose in (**B**) with that following the MD simulation (**C**) revealed that the bound ligand re-oriented, abandoning its initial Tet#1 interaction with Arg685 and establishing new stabilizing interactions with Arg residues 682 and 683 via ionic pi–cation bonding mechanisms (blue lines). Additional drug–receptor bond types included hydrophobic (green lines) and pi–pi (red lines) interactions. (**D**) Magnified view of the conformational pose of bisartan ACC519TT in the S2′ FCS pocket following VINA docking (see Methods). Details of the ligand–receptor interactions in the S2′ site are shown to the right (blue arrow). Color key: thin colored lines = primary intermolecular interactions; green = hydrophobic interactions; red = pi–pi; magenta = ionic; blue = pi–cation. Abbreviations: ACC519TT, benzimidazole *bis*-*N,N′*-biphenyltetrazole; Ala, alanine; Arg, arginine; Cys, cysteine; FCS, furin cleavage site; Ile, isoleucine; Gln, glutamine; Glu, glutamic acid; Gly, glycine; His, histidine; Leu, leucine; Lys, lysine; Phe, phenylalanine; Pro, proline; RMSD, root-mean-standard deviation; S, subunit; SARS-CoV-2, severe acute respiratory syndrome coronavirus 2; Ser, serine; Tet, tetrazole; Thr, threonine; Tyr, tyrosine.

Here, we describe molecular docking and MD results (Figure 4) indicating that the anionic bisartan ACC519TT undergoes stable binding to the S1/S2 and S2′ domains; thus, it may have the potential to block furin access to both FCSs. For example, in a ~90 ns MD simulation (NPT ensemble, 311 °K, 0.9wt% NaCl, pH 7.4), the ACC519TT tetrazole#1 functional group underwent re-orientation from its initial (t = 0.0 ns) VINA-docked conformation in the S1/S2 domain to coordinate with one or more of the three proximate cationic Arg residues (i.e., Arg682, Arg683, and Arg 685) comprising a portion of the FCS loop consensus sequence (see Figure 4). Intermolecular interactions between the Arg residues and tetrazole were primarily of the pi–cation form. Prior to about 18.2 ns, tetrazole#1 mainly interacted with Arg685. However, by the end of the simulation at about 90 ns, this same tetrazole group had re-oriented to coordinate with residues Arg682 and Agr683 via pi–cation interactions (blue lines in Figure 4). The last frame of the MD simulation (90 ns) shows the stabilizing intermolecular interactions between bound ACC519TT and key residues comprising the S1/S2 domain. The ligand was further stabilized by hydrophobic (green lines) and pi–pi interactions (red and magenta lines) between the two tyrosine residues (i.e., Tyr660 and Tyr695) that bracketed the benzene ring of the central benzimidazole group.

Compound ACC519TT also docked strongly to the S2′ furin cleavage site of SARS-CoV-2 (11.98 kcal/mol; see right-hand lower panel of Figure 4). The ACC519TT binding mechanism at the S2′ site involved pi–pi interactions between tetrazole#2 (Tet#2) and His1058 and pi–pi (red lines) and ionic pi–cation interactions (blue lines) between Arg815 and tetrazole#1 (Tet#1). Stabilizing hydrophobic interactions and additional pi–pi bonding interactions were observed between the imidazole aromatic ring and Phe833 (red lines), as well as between the backbone biphenyls and Phe823, Ile870, Phe782, Ala831, Gly1059, and Thr778.

### 3.5. Docking Comparisons of Approved Influenza Drugs and Biphenyl Tetrazole Ligands Against Influenza Neuraminidase (NA)

Influenza virus possesses two major surface glycoproteins, including hemagglutinin (HA) and NA [43]. Both proteins elicit antigenic responses, which are used to track and delineate specific influenza strains. HA binds to sialic acid residues of receptors on the surfaces of host respiratory epithelial cells, thereby mediating virion adhesion and subsequent penetration into the cell by endocytosis. However, NA, a tetrameric protein that cleaves α-ketosidic bonds between sialic acid and adjacent sugars, is required for the release of newly replicated virions. Therefore, lacking NA participation, only a single round of viral replication can occur, severely restricting the infection process. NA in particular has been long recognized as a promising target for drug discovery [65]. NA inhibitors (NAIs) comprise the largest group of anti-influenza drugs and are currently the most prescribed drugs for the treatment of human influenza [66,67]. Since the first NAI was approved by the FDA in 1999, several new anti-influenza drugs have been introduced and received FDA approval, including Tamiflu (oseltamivir), zanamivir, peramivir, and laninamivir, all of which are potent NAIs.

**Figure 5:**
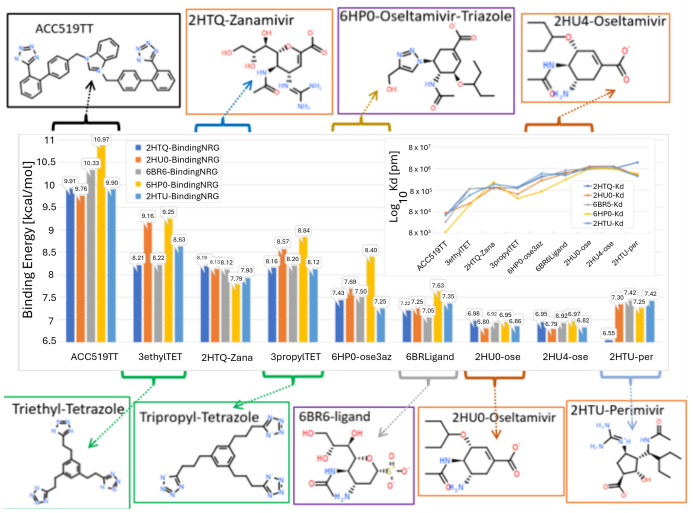
Docking of three FDA-approved drugs (orange borders), experimental drugs (purple borders), theoretical tri-tetrazole compounds (green borders), and our imidazole-biphenyltetrazole, ACC519TT (black border) to five influenza neuraminidases from the PDB. The four-letter name prefixes indicate the PDB complex from which the ligand was extracted prior to docking to the Arg-rich catalytic pocket of the apo-receptor. The number of AutoDock VINA runs per ligand ranged from 100 to 300 using AMBER14 charges and parameters. Abbreviations: ACC519TT, benzimidazole *bis*-*N,N′*-biphenyltetrazole; AMBER, Another Model Building Energy Refinement; Arg, arginine; PDB, Protein Data Bank. Chemical key: H, hydrogen; N, nitrogen; O, oxygen; S, sulfur.

**Figure 6:**
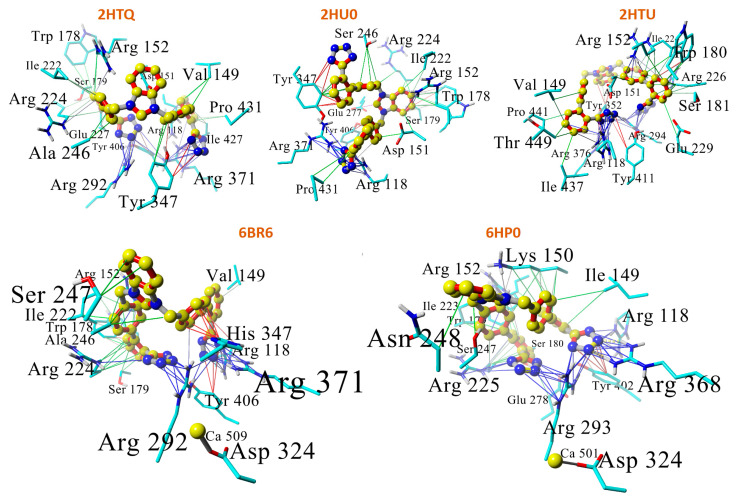
Mechanism of bisartan ACC519TT interactions with residues comprising the different neuraminidase catalytic domains. In four of the five neuraminidase models (i.e., 2HTQ, 6BR6, 6HP0, and 2HTU) both anionic tetrazole groups of the bisartan formed strong salt bridges (blue lines = cation–pi interactions) with two or more cationic arginine residues (carbon represented by yellow spheres). In the case of 2HU0, only one of the terminal tetrazole groups formed bonds with arginine residues (R371 and R118). The other tetrazole group formed pi–pi (red lines) and hydrophobic (green lines) interactions with Tyr347. In all the cases, the ligand displayed a “wrapped” conformation in which the tetrazole moieties were docked into positions relatively close to one another. Abbreviations: ACC519TT, benzimidazole *bis*-*N,N′*-biphenyltetrazole; Ala, alanine; Arg, arginine; Asn, asparagine; Asp, aspartate; Glu, glutamic acid; Ile, isoleucine; Lys, lysine; Pro, proline; Ser, serine; Thr, threonine; Trp, tryptophan; Tyr, tyrosine; Val, valine.

Eight drugs were docked to five different influenza NA crystallographic entries (PDB: 6BR6, 2HU0, 2HTQ, 2HTU, and 6HP0 (Figure 5)). The drugs included ACC519TT, three FDA-approved influenza drugs (i.e., zanamivir, oseltamivir, and peramivir), two experimental influenza drugs (i.e., 6BR6-ligand and an oseltamivir-triazole derivative), and two hypothetical tri-phenyltetrazoles. The PDB IDs correspond to the following NAs: (i) N8 NA in complex with zaramivir; (ii) N8 NA in complex with peramivir; (iii) N1 NA in complex with oseltamivir 1; (iv) N1 NA in complex with oseltamivir 2; (v) complex of NA from H1N1 influenza virus in complex with oseltamivir-triazole derivative; and (vi) N2 NA in complex with a novel antiviral compound. The docking results indicated that the bisartan ACC519TT exhibited the strongest docking to the Arg-rich NA catalytic domain compared to any of the other drugs tested (Figure 5). The mechanism of ACC519TT interactions with the different NA catalytic domains of the various NA models is characterized by the formation of strong salt bridges between the anionic tetrazole groups of the bisartan with two or more cationic arginine residues (Figure 6).

**Figure 7:**
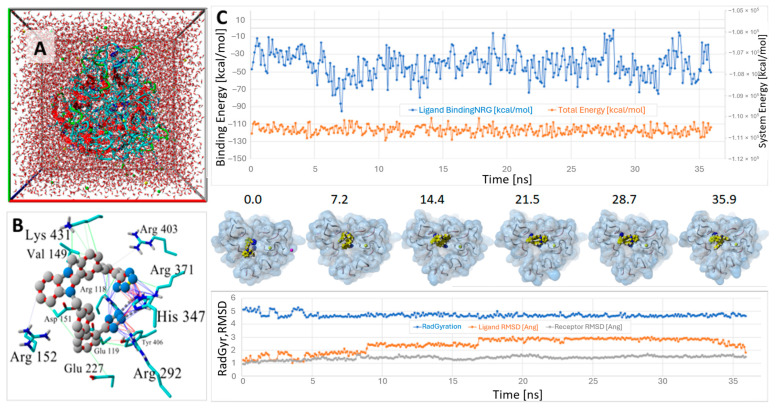
Equilibrium MD of ACC519TT docked in the neuraminidase 6BR6 catalytic site (NVT ensemble, 311 °K, 0.9 wt/% NaCl, pH 7.4). (**A**) Cuboid periodic cell with boundaries = 8.0 Å from any protein atom (Na and Cl atoms are shown as yellow and green balls in solution). (**B**) Docked ACC519TT (gray carbon atoms) at t = 0 ns showing main interactions with 6BR6 receptor (Key: green lines = hydrophobic; blue lines = salt bridge [cation–pi]; and red lines = pi–pi). (**C**) **Upper panel:** Ligand binding energy in kcal/mol (blue line); note that higher values = stronger binding to the receptor. Orange line = overall system potential energy. **Middle panel:** Frame captures of the ligand–receptor complex at designated intervals (ns). **Lower panel:** Ligand radius of gyration, RMSD (blue and orange lines, respectively), and receptor RMSD (gray line). The ligand remained stably bound in the pocket for the duration of the 36 ns run simulation. This stability was reflected in the relatively consistent ligand–receptor binding energy (blue line, upper panel). Abbreviations: ACC519TT, benzimidazole *bis*-*N,N′*-biphenyltetrazole; Arg, arginine; Asp, aspartic acid; Glu, glutamic acid; His, histidine; Lys, lysine; MD, molecular dynamics; RMSD, root-mean-standard deviation; Tyr, tyrosine; Val, valine; Å, angstrom.

**Figure 8:**
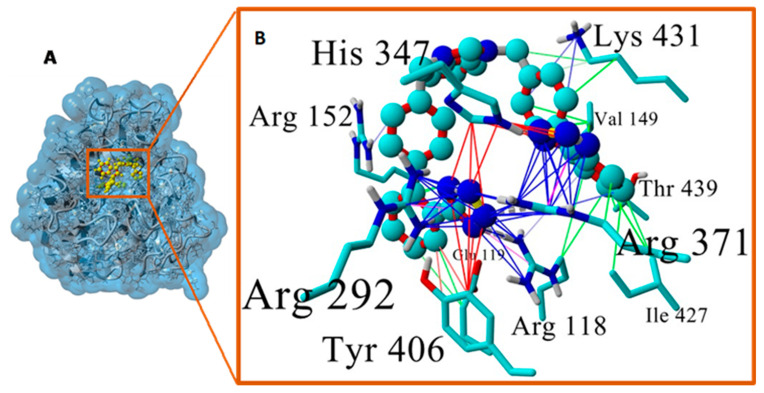
(**A**) Minimum-energy ACC519TT–neuraminidase complex (at about 10.5 ns) extracted from the 36 ns MD trajectory of 6BR6. Blue transparent shading corresponds to the 6BR6 water-accessible surface. (**B**) The anionic tetrazole groups form stable salt-bridge (cation–pi) interactions (blue lines) involving R118, R292, and R371. These are the same three Arg residues that were involved in bonding with the dual anionic tetrazole groups of ACC519TT in the original docked configuration at t = 0 ns. Abbreviations: ACC519TT, benzimidazole *bis*-*N,N′*-biphenyltetrazole; Arg, arginine; Glu, glutamic acid; Ile, isoleucine; His, histidine; Lys, lysine; MD, molecular dynamics; Thr, threonine; Tyr, tyrosine; Val, valine.

The docked ACC519TT-6BR6/NA complex was stable for the duration of a 36 ns NPT molecular dynamics simulation (NPT ensemble, 311 °K, 0.9 wt/% NaCl, pH 7.4; see Figure 7 and Figure 8). ACC519TT binding inside the catalytic domain of 6BR6 (and other NA receptors) were mainly stabilized by cation–pi (salt bridge)-type interactions between the anionic tetrazole groups with cationic arginine residues R118, R292, and R371.

**Figure 9:**
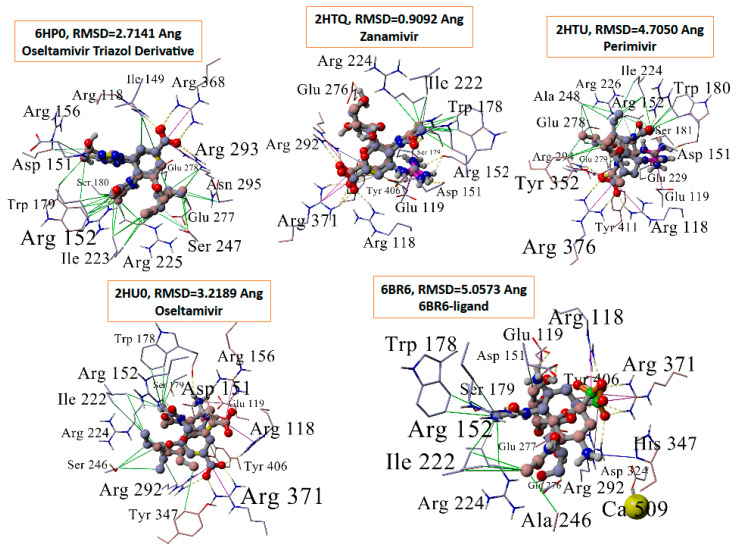
Re-docking of extracted native ligands into the catalytic pocket of the five neuraminidase models evaluated. In each case, the X-ray crystallographic structure (dusty blue carbon atoms) was superimposed against the docked complex (maroon carbon atoms) before calculating the RMSD values for the superimposed ligands. Docking to 6HP0 (RMSD = 2.7141 Å) and 2HTQ (RMSD = 0.9092 Å) yielded the best fit with their respective X-ray conformations, whereas poorer-quality fits were observed for PDB 2HU0 (RMSD = 3.2189 Å), 6BR6 (RMSD = 5.0573 Å), and 2HTU (RMSD = 4.7050 Ang). Abbreviations: Ala, alanine; Arg, arginine; Asn, asparagine; Asp, aspartic acid; His, histidine; Ile, isoleucine; Glu, glutamic acid; PDB, Protein Data Bank; RMSD, root-mean-square deviation; Ser, serine, Trp, tryptophan, Tyr, tyrosine; Å, angstrom.

The minimum-energy ACC519TT-6BR6/NA complex (at about 10.5 ns) extracted from the 36 ns MD trajectory is shown in Figure 8. The binding mechanism of ACC519TT was dominated by pi–cation (ionic salt bridge) interactions with Arg residues. The re-docking of the extracted native ligands into the catalytic pocket of the four NA models was performed. In each case, the X-ray crystallographic structure was superimposed against the docked complex before calculating the RMSD values for the superimposed ligands (Figure 9).

### 3.6. VINA Binding Energy and Per-Atom Efficiency Comparisons for Ten Drugs Against Three Distinct NA Receptors

**Figure 10:**
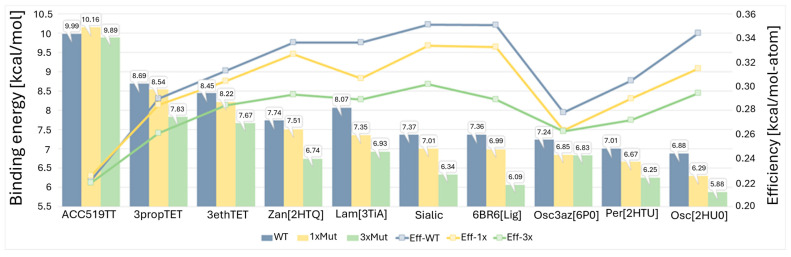
Docking data comparing VINA binding energies and per-atom efficiencies for 10 drugs against three different neuraminidase receptors: neuraminidase in complex with sialic acid, its single mutant 1×Mut (R153A), and the triple mutant 3×Mut (R153A-R294A-R372A).

Furthermore, we conducted a comprehensive comparison of VINA binding energies and per-atom efficiencies for ten drugs against three distinct neuraminidase receptors (Figure 10). The ten drugs under scrutiny encompassed a diverse array of antivirals: (1) ACC519TT—an experimentally validated bisartan antiviral (Figure 1C, Figure 3F and Figure 5); (2) tripropyl-tetrazole—a theoretically proposed drug [68]; (3) triethyl-tetrazole—a theoretically proposed drug [69]; (4) zanamivir—an FDA-approved antiviral specifically targeting influenza neuraminidase [70]; (5) laninamivir—an FDA-approved antiviral specifically targeting influenza neuraminidase [71]; (6) sialic acid—the native substrate for neuraminidase [72]; (7) 6BR6[Lig]—an experimentally validated neuraminidase inhibitor extracted from PBD:6BR6 [73]; (8) oseltamivir-triazole—an experimentally validated neuraminidase inhibitor extracted from PDB:6HP0 [74,75] (9); peramivir—an FDA-approved antiviral specifically targeting influenza neuraminidase extracted from PDB:2HTU [76]; and (10) oseltamivir—an FDA-approved antiviral specifically targeting influenza neuraminidase extracted from PDB:2HU0 [55]. The neuraminidase receptors utilized in the study include the crystal structure of neuraminidase in complex with sialic acid (PDB:5L18), its single mutant 1xMut (R153A), and the triple mutant 3xMut (R153A-R294A-R372A). To establish these mutants, alanine substitutions were introduced at specific pocket loci deemed crucial based on a paper by Gubareva et al. [77].

Our observations revealed that ACC519TT exhibited remarkable resilience to mutations when compared to the other drugs. Notably, the binding affinity of ACC519TT remained conspicuously high, irrespective of the presence or absence of mutations. It is essential to underscore, however, that the relatively larger size of ACC519TT, in contrast to the smaller drugs, engendered a lower per-atom binding efficiency. This phenomenon warrants careful consideration, as it may have implications for the stability and dynamics of ACC519TT during MD simulations, which requires further rigorous examination (to be investigated). In stark contrast to ACC519TT, the binding of all other drugs, except ACC519TT, to the single-mutant receptor (R153A) or the triple-mutant receptor (R153A-R294A-R372A) was substantially attenuated when compared to the wild-type receptor. Furthermore, the binding of all drugs to the triple-mutant receptor exhibited a reduced affinity compared to the single-mutation receptor.

## 4. Discussion

Herein, we evaluated the pan-antiviral ability of our novel bisartan, ACC519TT, in comparison to other commercially available sartans and antivirals. The work presented in this study delves into an extensive investigation of bisartans, a novel class of highly potent sartans, with a focus on their potential as pan-antiviral drugs. Bisartans, bisalkylated imidazole/benzimidazole sartans bearing dual symmetric anionic biphenyl tetrazole moieties, are the result of pioneering research on losartan, which led their discovery [43,47]. This research sheds light on the mechanism of the AngII action at the AngII-type 1 receptor (AT1R) found to be relevant to the charge relay system in Ser proteases [48]. The conformation of AngII, the principal effector of the RAS, was extensively investigated based on structure–activity relationship studies, nuclear magnetic resonance fluorescence, and molecular modeling techniques [78].

This study has been undertaken with the knowledge that CVD is related to COVID-19 in terms of the mechanisms that trigger disease initiation and progression [79]. Through the utilization of MD simulations, this work aims to elucidate the ability of bisartans to target commonalities shared among three significant viruses: SARS-CoV-2, influenza, and RSV. This investigation is crucial for the development of a broad-spectrum antiviral treatment that can effectively combat multiple viral infections. Overall, the comprehensive findings from this study provide a substantial contribution to the field of antiviral research. The exceptional binding affinity demonstrated by ACC519TT toward various receptor domains common to multiple viruses, including NSP3, NA, and RSV F-protein, underscores its potential as a versatile and potent therapeutic option. Moreover, the stability of the ACC519TT–viral protein complexes observed through molecular dynamics simulations provides valuable insights into its efficacy as a stable inhibitor. These significant outcomes contribute to the ongoing scientific efforts aimed at developing effective pan-antiviral treatments to combat viral pandemics.

ACE2 is involved in the early stages of COVID-19, as the binding of the S-protein to the enzyme is the first step of infection. A detailed review summarized the findings of several in silico-based studies of antiviral drugs, which bind in the interface between ACE2 and the S-protein [80]. Investigations of several sartans and bisartans were undertaken to determine if these compounds (i) bind to ACE2, (ii) inhibit the ACE2 enzyme catalysis of AngII to angiotensin (1–7), and (iii) inhibit the binding of COVID-19 S-protein to ACE2. Studies involving surface plasmon resonance binding to ACE2 have shown that of the sartans/bisartans investigated, benzimidazole-based bisartans bind more strongly to ACE2 (dissociation constants in the nM range) compared to imidazole-based sartans [18]. Current findings suggest that the mechanism of action of sartans is intracellular, probably by inhibiting a processing enzyme required for virion replication [81].

The varying binding affinities and per-atom efficiencies observed for the ten drugs against different NA receptor mutants underscore the significance of receptor conformational changes and ligand flexibility in drug–receptor interactions. These findings offer valuable insights into the molecular underpinnings of drug efficacy and may have implications for rational drug design and optimization.

The exceptional resilience of ACC519TT to mutations could be attributed to its higher degrees of freedom of motion, stemming from a greater number of torsion bonds relative to the other drugs. This inherent flexibility enables ACC519TT to efficiently adapt and interact with the binding pocket, even when a single arginine residue remains within it. Consequently, ACC519TT can undergo interactions with one or both of its tetrazole groups, thereby maintaining its binding affinity despite the presence of mutations.

Overall, bisartan ACC519TT bearing two biphenyl tetrazoles on a benzimidazole scaffold was found to be the best binder compared to known inhibitors of the three viruses under investigation. ACC519TT was a stronger binder compared to the FDA-approved drugs zanamivir, oseltamivir, and peramivir against influenza virus NA protein. The two tetrazoles were interrupting the internal interactions between sialic acid and Args, which stabilized the conformation of glycoprotein NA, thus inhibiting its virion action. ACC519 TT was also a stronger binder compared to crystal structure experimental NSP3 Mac1 inhibitors R1104 and R7335, which both contain anionic tetrazole and fused pyridine-like aromatic rings essential for pi–pi contacts. ACC519TT was also a stronger binder compared to RSV inhibitors Cpd2-RSV and Cpd44-RSV, which complex with the RSV F-protein due to the two tetrazoles and the strong aromatic character of the benzimidazole and of the two biphenyl groups, increasing the interactions and affinity with the protein domains of the three viruses. ACC519TT was, furthermore, an excellent binder of furin, preventing the cleavage of furin cleavage sites S1/S2 and S2′, which initiate infection by complexing with the Args of the cleavage sites, thus preventing disease.

### 4.1. The Role of Tetrazoles and the Unique Properties of Bisartans

Pioneer research on the design and synthesis of losartan analogs has led to the discovery of a new class of ARBs with N mono biphenyl tetrazole substitution and the butyl group at position 5 of the imidazole scaffold [82,83]. These analogs were the basis to further develop bisalkylated derivatives bearing two biphenyl tetrazole groups on the two imidazole nitrogens, called bisartans [15,82,83]. They are *N,N′* symmetrically bis-substituted 5 butyl imidazole analogs with notable properties [82,83]. Bisartans are unique molecules that contain two tetrazoles that strongly interact with positive Args and form pi–cation interactions with aromatic rings. Tetrazole is a bioisoester of carboxylic acid, metabolically more stable to many biological transformations that the functionality of carboxylic acid is susceptible to in the liver. Moreover, tetrazole has the capacity to exert various non-covalent interactions with biological targets, and its derivatives possess diverse pharmacological properties of great interest in recent years in several diseases [84]. The presence of two tetrazoles within the same molecule and the imidazole/benzimidazole scaffold of bisartans make them and, in particular, ACC519TT, superior over the known inhibitors of the three viruses. Extensive structure–activity studies focusing on the role of the two tetrazoles and the benzimidazole scaffold are essential for understanding the strong pi–pi interactions that make ACC519TT superior to the known inhibitors of the three viruses. These studies can provide valuable insights into the molecular interactions and binding mechanisms that contribute to ACC519TT’s enhanced antiviral activity against these viruses. By elucidating the specific structural features responsible for its potency, researchers can further optimize and design novel inhibitors with even greater efficacy, potentially opening new avenues for antiviral drug development and therapeutic interventions. Conducting rapid toxicological assessments of the potential antiviral drugs is equally crucial. These assessments should include an exploration of drug-induced immunosuppression [85], which has the potential to foster opportunistic infections. These meticulous procedures ensure that emerging treatments not only are efficacious but also secure against viral infections.

### 4.2. Promising Directions for Further Exploration in the Utilization of Bisartans

Patients who already have a CVD appear to be more likely to contract COVID-19 and typically have a more severe disease course with poorer clinical outcomes [86,87,88,89]. Although this cohort’s prevalence of diabetes and hypertension was comparable to that of the general Chinese population, it was noticeably greater than that of cardio-cerebrovascular disease. More significantly, having diabetes, CVDs, or hypertension increased the likelihood of having a severe illness or needing intensive care unit admission by two-, three-, and two-fold, respectively. This suggests that these comorbidities influence prognosis. Clinical outcomes in 44,672 confirmed cases of COVID-19 were documented in a larger publication from the Chinese Center for Disease Control and Prevention. The overall case fatality rate for the entire group was 2.3%, although it was noticeably higher in patients with hypertension, diabetes, and CVDs (6%, 7.3%, and 10.5%, respectively) [86]. Cancer and CVDs, including ischemic myocardial injury, heart failure, and other cardiovascular problems owing to diabetes, chronic renal impairment, and hypertension, have been linked to higher AngII levels. Inflammation, the epithelial-to-mesenchymal transition, and unfavorable vascular network remodeling are some of the shared AngII-dependent etiopathological pathways that cardiovascular and cancer share despite their obvious phenotypic distinctions [90]. It is known that pre-existing CVDs may increase the severity of COVID-19 through an AngII-dependent mechanism because ACE2 is found on the surface of various cells, including those in the respiratory tract and cardiovascular system. The use of “bisartans” in the treatment of diabetes, CVDs, and hypertension may be advantageous [15]. Through in silico molecular modeling and ex vivo rabbit vascular tests, preliminary research has demonstrated the ability of bisartans to strongly antagonize the AT1R [91]. In order to increase salt-bridge interactions with the AT1R residuals Arg167 and Lys199, bisartans are synthesized to have a core comprising bis-alkylated imidazole with two symmetrically anionic tetrazole groups. This results in overwhelming, implacable antagonistic interactions that cannot be overcome by raising the concentration of AngII [92]. In addition to the aforementioned properties, we have recently published a study that evaluated the ability of our nirmatrelvir, imidazole, and benzimidazole bisartans (i.e., ACC519TT, benzimidazole-*N*-biphenyltetrazole, and 4-butyl-N,N0-*bis* [20-2Htetrazol-5-yl)biphenyl-4-yl]methyl)imidazolium bromide) and commonly prescribed sartans (i.e., candesartan and telmisartan) to reduce contraction responses in isolated iliac arteries from male New Zealand White rabbits to various vasopressor substances (e.g., angiotensin A, AngII, and phenylephrine). Importantly, this study showed the inability of nirmatrelvir to reduce contraction responses to AngII, angiotensin A, and phenylephrine; however, our bisartans, including ACC519TT, were able to insurmountably inhibit AngII- and angiotensin A-mediated contraction even at ultra-high dilutions (i.e., 10^−60^ M), a phenomenon interpreted by the entangled quantum pharmacology mechanism [35]. Thus, our bisartans may be superior to other antivirals, as not only do they prevent infectivity, but they also have cardiovascular protective effects, an ability that is not shared by nirmatrelvir. Further research, including clinical trials, is essential to evaluate the safety and efficacy of bisartans in treating CVDs.

### 4.3. Challenges and Limitations Associated with Using Bisartans as Anti-SARS-CoV-2 Agents

The use of bisartans as potential anti-SARS-CoV-2 medicines draws attention to a variety of noticeable restrictions and difficulties. The most important of these factors is the requirement for a thorough evaluation of their safety and toxicity profiles in the context of treating COVID-19, with a focus on potential side effects resulting from prolonged administration. To conclusively establish both the safety and efficacy of bisartans, it is essential to perform rigorous in vivo animal experiments and human clinical trials, a complicated task that necessitates significant time and resource commitments. Concerns about possible therapeutic interactions, the development of latent virus resistance, and the painstaking selection of an ideal dosage schedule all arise concurrently. A significant logistical difficulty is presented by navigating the complex world of regulatory clearances, as well as the dilemma of establishing a strong drug supply chain and evenly dispersed access during a pandemic. It is important to give serious thought to the financial aspects of drug development, as well as affordability issues for a wide range of patient populations. Furthermore, rigorous validation is required to understand the precise molecular underpinnings defining the activity of bisartans in suppressing SARS-CoV-2. This mandate for validation includes knowledge of the long-term effects of bisartan use, the variation in therapeutic responses between patients, and the moral considerations guiding its application within the COVID-19 therapeutic framework.

In summary, Args play a significant role in the ligand–protein interactions in many biological systems, as they stabilize conformations and bind strongly to anionic sites. Anionic sartans and, in particular, bisartans are stronger binders that are able to neutralize cationic Args, thus protecting the biological systems from disease processes when they are mediated by Args, as in the SARS-CoV-2 spike protein cleavage by furin. Additional synthetic and physicochemical methods could help resolve and confirm predicted computational interactions involving arginine and microwave solid-phase peptide synthesis [93]. Tetrazole-containing compounds like sartans and bisartans that block Args are ideal candidate drugs to battle arginine-based infections [15,16,17,34].

## 5. Conclusions

Bisartans, characterized by the presence of two biphenyl tetrazoles as pharmacophore warheads on imidazole or benzimidazole scaffolds, exhibit remarkable affinity and strong binding to the catalytic sites of the three viruses. Notably, when compared to other antivirals, bisartan ACC519TT and its analogs (especially the cyano-derivatives) displayed equivalent or stronger binding to NSP3, PLpro, NA, and the S1/S2 cleavage site and S2′ cleavage site of furin (proteins essential for the replication, infectivity, and entry into host cells of multiple viruses, including SARS-CoV-2, influenza, and RSV). Thus, the putative ability of our newly synthesized bisartans to inhibit SARS-CoV-2 entry, target multiple viruses, and potently inhibit influenza NA and RSV F-protein highlights them as versatile, broad-spectrum candidates for viral treatment. Moreover, their role in the RAS (i.e., activating ACE2 and converting Ang II to angiotensin 1–7) highlights their potential in mitigating the cytokine storm associated with COVID-19, as well as CVDs. It is essential to recognize that the insights obtained from this study provide a foundation for further investigations into the structural and dynamic aspects of drug–receptor interactions. Future research endeavors could leverage computational simulations and experimental validation to gain a deeper understanding of the intricacies governing ligand binding and recognition. Ultimately, these insights could pave the way for the development of novel and more efficacious antiviral therapeutics.

## Figures and Tables

**Table 1:** Inhibitors of SARS-CoV-2, influenza, and respiratory syncytial virus.

Inhibitor Type	SARS-CoV-2 Inhibitors	Influenza Inhibitors	RSV Fusion(F)-Protein Inhibitors
ACE2 inhibitors	Sartan-bound ACE inhibitors		
Furin inhibitors	Dichloropyridine-based inhibitors		
NA (Arg-rich) inhibitors		Zanamivir, oseltamivir, and peramivir	
NSP3 Mac1 inhibitors	Benzimidazole-based inhibitors (i.e., R1104 and R7335)		Benzimidazole-based inhibitors (i.e., Cpd2-RSV and Cpd44-RSV) and tetrazole inhibitors
S1/S2 and S2′ cleavage site blockers	Arg blockers (i.e., bisartans and sartans)		
3CLpro inhibitors	Paxlovid and nirmatrelvir (active antiviral ingredient)		
RSV fusion inhibitors Key amino acids (R339, F137, F140, and F488)			Cpd2, Cpd44, and tetrazole compounds

Abbreviations: ACE, angiotensin-converting enzyme; Arg, arginine; Cpd, compound; F-protein, fusion protein; Mac1, macrodomain-1; NA, neuraminidase; NSP3, non-structural protein 3; RSV, respiratory syncytial virus; S, subunit; 3CLpro, 3-chymotrypsin like protease.

## Data Availability

Ligand–receptor docking was performed using AutoDock VINA and MD simulations were conducted using the YASARA Dynamics software: http://www.yasara.org/ (accessed 1 May 2024). The datasets generated and analyzed during the current study are available on the Zenodo platform, which can be retrieved using the following link: https://zenodo.org/records/12597147 (accessed 30 June 2024).
